# Preoperative ultrasound-guided dual localization with titanium clips and carbon nanoparticles for predicting the surgical approach and guiding the resection of Siewert type II esophagogastric junction adenocarcinoma

**DOI:** 10.1007/s00432-024-05689-3

**Published:** 2024-03-20

**Authors:** Weihang Wu, Ziqiang Luo, Yongchao Fang, Li Yu, Nan Lin, Jin Yang, Hu Zhao, Chunhong Xiao, Yu Wang

**Affiliations:** 1 Department of General Surgery, Fuzong Clinical Medical College of Fujian Medical University, 900th Hospital of Joint Logistics Support Force, PLA, Fuzhou, China; 2 Department of General Surgery, Dongfang Affiliated Hospital of Xiamen University, School of Medicine, Xiamen University, Xiamen, China; 3 Department of Gastroenterology, Fuzong Clinical Medical College of Fujian Medical University, 900th Hospital of Joint Logistics Support Force, PLA, Fuzhou, China

**Keywords:** Adenocarcinoma of the esophagogastric junction, Carbon nanoparticle, Titanium clip, Endoscopic ultrasonography, Surgical pathway

## Abstract

**Objective:**

To investigate the superiority of preoperative ultrasound-guided titanium clip and nanocarbon dual localization over traditional methods for determining the surgical approach and guiding resection of Siewert type II adenocarcinoma of the esophagogastric junction (AEG).

**Method:**

This study included 66 patients with Siewert type II AEG who were treated at the PLA Joint Logistics Support Force 900th Hospital between September 1, 2021, and September 1, 2023. They were randomly divided into an experimental group (*n* = 33), in which resection was guided by the dual localization technique, and the routine group (*n* = 33), in which the localization technique was not used. Surgical approach predictions, proximal esophageal resection lengths, pathological features, and the occurrence of complications were compared between the groups.

**Result:**

The use of the dual localization technique resulted in higher accuracy in predicting the surgical approach (96.8% vs. 75.9%, *P* = 0.02) and shorter proximal esophageal resection lengths (2.39 ± 0.28 cm vs. 2.86 ± 0.39 cm, *P* < 0.001) in the experimental group as compared to the routine group, while there was no significant difference in the incidence of postoperative complications (22.59% vs. 24.14%, *P* = 0.88).

**Conclusion:**

Preoperative dual localization with titanium clips and carbon nanoparticles is significantly superior to traditional methods and can reliably delineate the actual infiltration boundaries of Siewert type II AEG, guide the surgical approach, and avoid excessive esophageal resection.

**Supplementary Information:**

The online version contains supplementary material available at 10.1007/s00432-024-05689-3.

## Introduction

Adenocarcinoma of the esophagogastric junction (AEG) refers to adenocarcinomas located within a range of 5 cm above and below the esophageal gastric junction (Siewert et al. 1987). AEG is an independent disease that is different from upper esophageal cancer and distal gastric cancer, and is divided into three types based on its location: Siewert type I refers to adenocarcinomas in which the tumor center is located within a range of 1–5 cm above the dentate line; Siewert type II, to adenocarcinomas located between 1 above and 2 cm below the dentate line; and Siewert type III, to adenocarcinomas located between 2 and 5 cm below the dentate line (Meng-xin and yanjunacang Shun-dong. 2021). The incidence rate of AEG has significantly increased worldwide (Manabe et al. 2022; Uhlenhopp et al. 2020). Currently, surgery is the only curative approach for AEG.

Surgical resection of AEG is mainly performed via the thoracic and abdominal approaches. Studies have shown that resection of Siewert type III AEG requires an abdominal approach, while Siewert type I tumor resection requires thoracotomy and lymph node dissection (D'journo X B. 2018). For Siewert type II AEG, surgeons often determine the surgical approach by combining the findings of preoperative gastroscopy, barium meal, CT, etc., with the location and extent of tumor invasion. Thoracic surgeons believe that a transthoracic approach can ensure sufficient length of the proximal margin, a negative upper margin, and effective mediastinal lymph node dissection (Peng et al. 2015), while gastrointestinal surgeons believe that the transabdominal approach can reduce the incidence of pulmonary complications and shorten postoperative hospital stay (Fei et al. 2021). Thus, the optimal surgical approach for Siewert type II AEG has always been controversial in clinical practice.

A key objective of AEG surgery is to ensure a negative surgical margin (Japanese gastric cancer treatment guidelines 2014), but the unique biological behavior of AEG causes cancer cells to infiltrate the esophageal side via the submucosal direction. With the routine staining and electronic endoscopy approach for localization, it is only possible to observe the lesion range on the mucosal surface. Therefore, it is impossible to determine whether the tumor has infiltrated the submucosal layer. In addition, as the staining time of commonly used staining agents, such as methylene blue, indigo carmine, indocyanine green, and other dyes, is relatively short, they can easily contaminate the surgical field over time and cause inaccurate localization (Price et al. 2000; Technology Committee et al. 2010; Takada et al. 2023; Nagami et al. 2018). If surgeons only determine the extent of tumor resection by manual observation or palpation during surgery, it is easy to overestimate the extent of resection of the esophageal length. This could lead to an increase in the risk of positive surgical margins, difficulty in reconstructing anastomoses, and increased surgical trauma, among other related problems.

The esophagus enters the abdominal cavity from the thoracic cavity at the esophageal hiatus, approximately at the level of the 10th thoracic vertebra (Kahrilas et al. 2008). Therefore, in the case of AEG lesions that invade the junction of the esophagus and stomach, the diaphragm may be involved. This might necessitate different surgical approaches. Alternatively, endoscopic ultrasonography (EUS) can be used to accurately detect the extent of submucosal infiltration of AEG and determine the extent of tumor invasion (Takamaru et al. 2020). Further, as an alternative staining agent, carbon nanoparticles have advantages such as stable properties and longer staining time compared to traditional staining agents, and no serious side effects have been reported yet (Wang et al. 2013). As a solution to overcoming these challenges associated with traditional approaches, this study explores preoperative EUS with carbon nanoparticle and titanium clip labeling as an alternative to traditional techniques for marking the tumor boundary and determining the extent of submucosal invasion of the tumor. In addition to EUS, abdominal radiography was used to determine the height of the titanium clip, and the surgical approach was predicted based on the relationship between the position of the titanium clip and the lower edge of the 10th thoracic vertebra. During surgery, the resection boundary was determined by palpating the titanium clip and observing the range of carbon nanoparticle black staining, to ensure a negative surgical margin.

## Patients and methods

### Patients

**Fig. 1:**
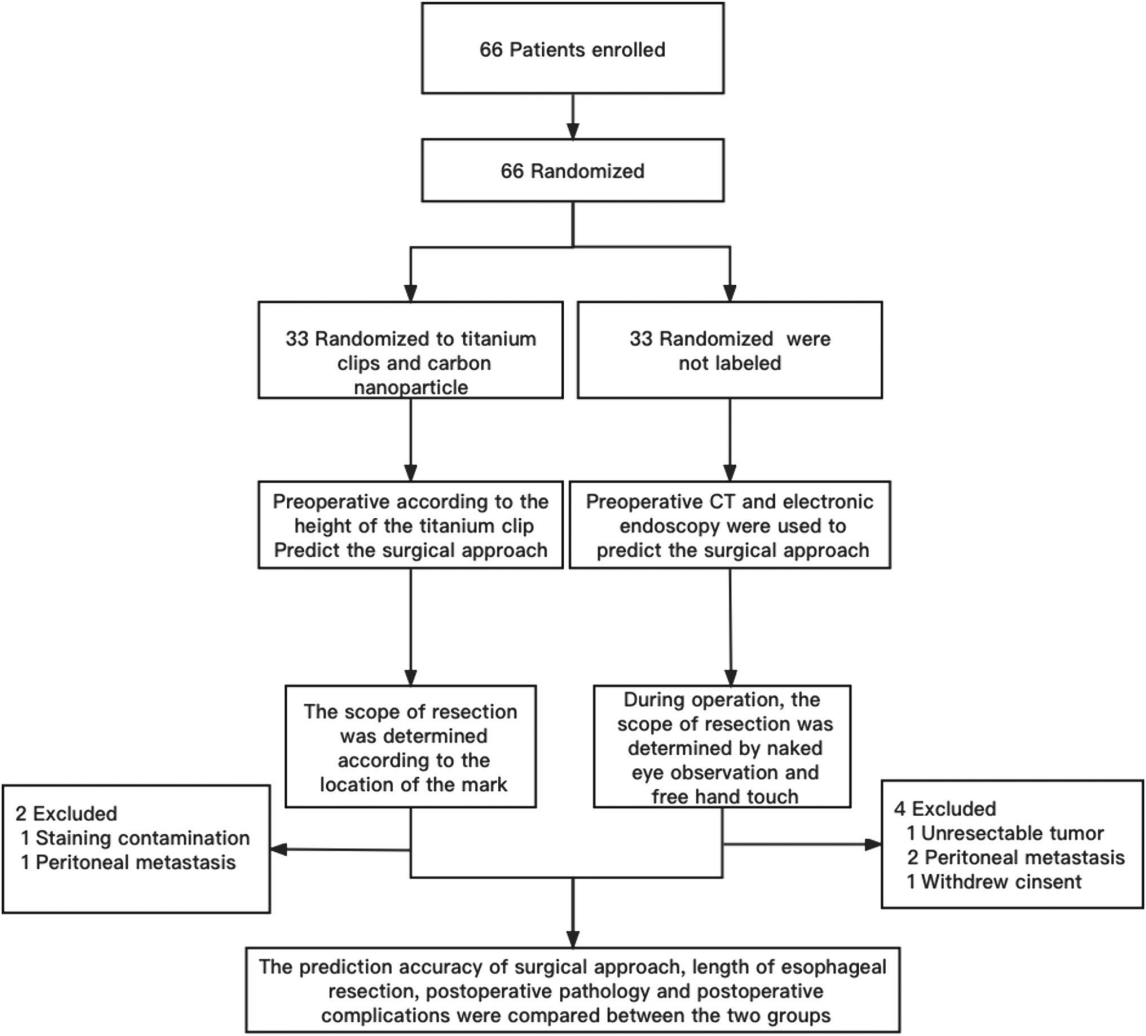
Flow chart depicting patient grouping, preoperative and perioperative assessments, postoperative exclusion criteria, and postoperative assessments

This study included patients who were diagnosed with AEG based on endoscopic biopsy and pathological examination at the Ninth Hospital of the Joint Logistics Support Force. Based on the findings of electronic gastroscopy, gastrointestinal barium meal, abdominal CT examination, electrocardiography, cardiac ultrasonography, lung function tests, and other examinations, a comprehensive evaluation was conducted to determine whether the patients were suitable for curative surgery. Only those who were eligible for surgical procedures were considered for the study. A total of 66 patients were selected and randomly divided into an experimental group and a routine group comprising 33 cases each. The inclusion criteria were as follows: (1) diagnosis of AEG based on the pathological results of endoscopic biopsy, (2) classification of AEG as Siewert type II, (3) diagnosis of cIA–IIA stage disease based on CT and EUS examination, (4) no history of other malignant tumors or tumor recurrence and ability to tolerate surgery based on the comprehensive evaluation mentioned earlier. The exclusion criteria were (1) diagnosis of cIIB–IV stage disease based on CT and EUS examination, (2) tumor metastasis that makes resection impossible, (3) staining contamination or detachment of carbon nanoparticle or titanium clip, (4) a history of spinal deformities or fractures, (5) poor cardiopulmonary function and resultant inability to tolerate surgical trauma, and (6) presence of combined malignant tumors. Figure 1 illustrates the patient flow through the trial.

### Ethics statement

This study was conducted in accordance with the Declaration of Helsinki and was approved by the Ethics Committee of the 900th Hospital of the Joint Logistics Support Force of the PLA (Approval no. 2021–047).

### Clinical staging and labeling

**Fig. 2:**
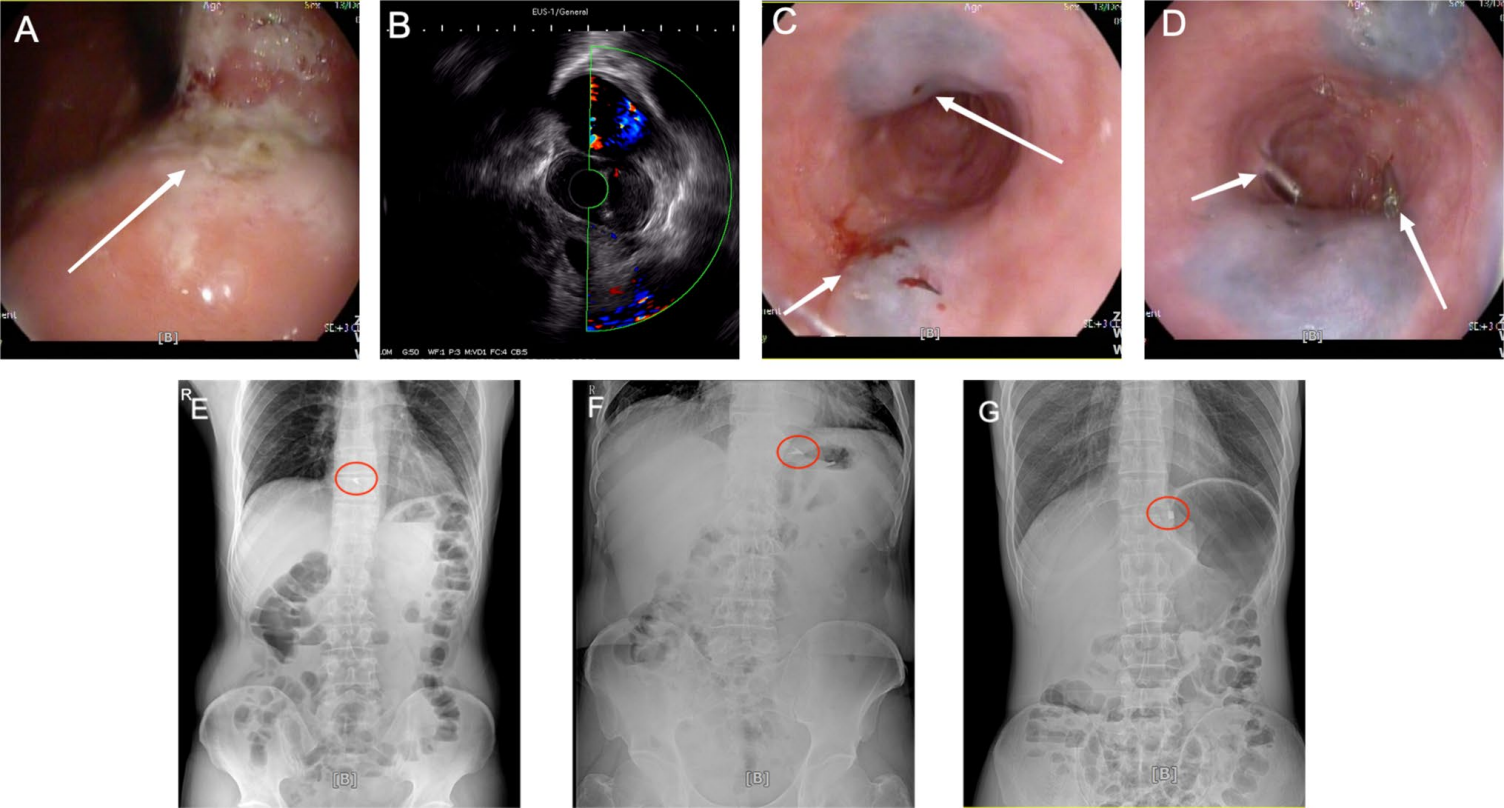
Images depicting the dual localization technique with titanium clips and carbon nanoparticle labeling. **A** Direct observation with endoscopic ultrasound. **B** Endoscopic ultrasound detection. **C** Image taken after completion of carbon nanoparticle labeling. **D** Image taken after placement of the titanium clips. **E** Radiograph showing the position of the titanium clip above the 10th thoracic vertebral body. **F** Radiograph showing the position of the titanium clip between the upper and lower edges of the 10th thoracic vertebral body. **G** The titanium clip is located below the 10th thoracic vertebral body

The preoperative clinical staging of the patients was done according to the 8th edition of the AJCC cancer staging system. After patients in the experimental group had undergone routine examinations such as electronic gastroscopy, pathological examination, gastrointestinal barium meal, and abdominal CT examination, dual localization with titanium clips and carbon nanoparticles was performed under direct EUS 1–2 days before surgery. The patient were fasted for at least 4–6 h before labeling. After induction of anesthesia, endoscopists perform EUS to examine the esophagus, esophagogastric junction, stomach, and surrounding lymph nodes. Through direct observation (Fig. 2A) and ultrasound detection (Fig. 2B), the actual depth of tumor infiltration and the actual height of invasion of the esophagus were determined. Two points located 1 cm from the oral side of the actual boundary of the tumor were selected, and 0.1 ml of a carbon nanoparticle suspension was injected into the protruding esophageal or gastric wall of the two points. Next, two additional points at the same height and on the same plane as the previous injection points were selected and marked with titanium clips. It was ensured that the carbon nanoparticle suspension was free of contamination, the injection point was free of bleeding, and the titanium clips were not dislodged (Fig. 2C and D). After the patient had woken up, abdominal radiography was performed to confirm the height of the titanium clip (Fig. 2E, F, and G). The routine group patients underwent routine examination with electronic gastroscopy, pathological examination, gastrointestinal barium meal, and abdominal CT examination, without the use of titanium clips and carbon nanoparticle positioning markers.

### Surgical protocol

**Fig. 3:**
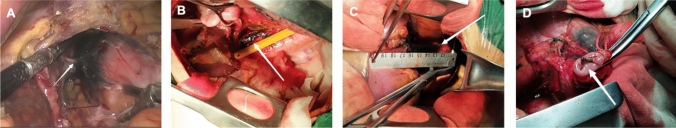
Images depicting the surgical approaches adopted for the resection procedures. **A** Transabdominal approach. **B** Combined thoracoabdominal approach. **C** Transthoracic approach. **D** Titanium clips

In the experimental group, the surgical approach was determined based on the positional relationship between the titanium clip in the abdominal standing plain radiograph and the 10th thoracic vertebra: a transthoracic approach (TTA) was adopted upstream of the 10th thoracic vertebra; a transabdominal approach (TAA), below the 10th thoracic vertebra; and a combined thoracoabdominal approach (CTA), between the upper and lower edges of the 10th thoracic vertebra. During the procedure, the resection range of the lesion was determined by palpating the titanium clip and observing the range of black staining with the carbon nanoparticles (Fig. 3A, B, C, and D).

In the routine group, after comprehensive evaluation based on abdominal radiography, abdominal CT examination, and other exams, either transabdominal surgery or transabdominal surgery was performed. If the surgery could not be completed due to difficulties in anastomosis or positive results for the intraoperative frozen pathological samples, combined thoracoabdominal surgery was performed. Lymph node dissection includes D2 lymph node resection, middle and lower mediastinal lymph node dissection, and bilateral lymph node resection (Moehler et al. 2015).

### Specimen processing

**Fig. 4:**
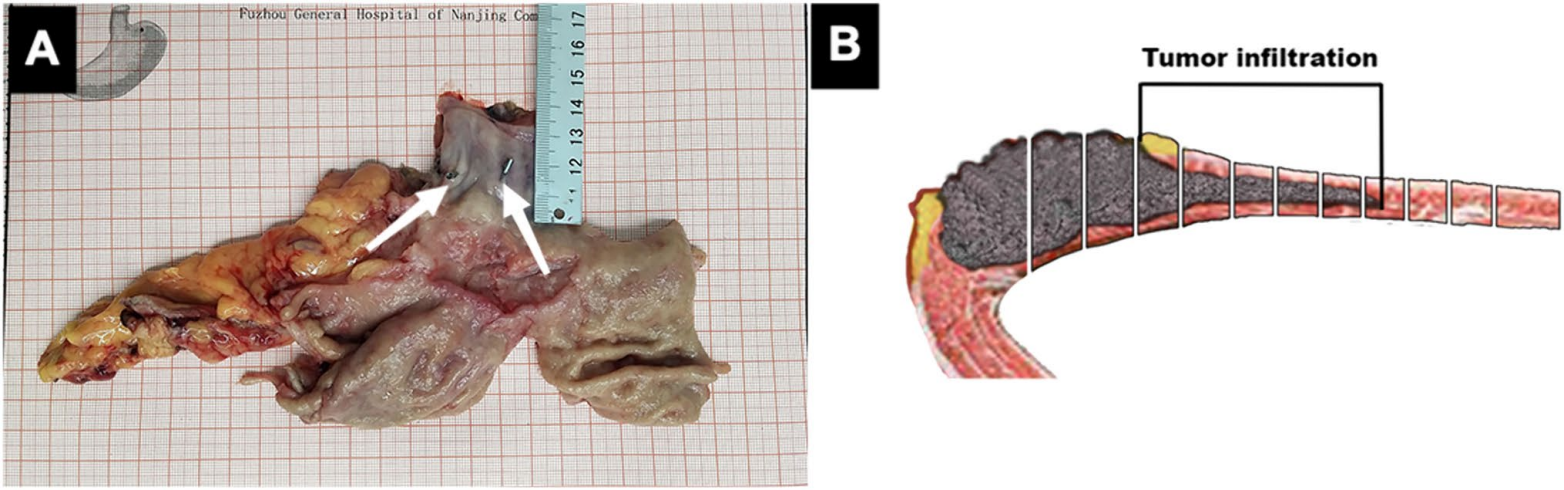
Measurement of the resected specimen and sampling of tissue for pathological slides. **A** Measurement of the length of the proximal esophageal area of the resected specimen and the titanium clips (indicated with arrows). **B** Diagram depicting the pathological sampling of the resected specimen

After the specimen was detached, it was cut along the greater curvature of the esophagus and stomach to keep a safe margin around the lesion and avoid cutting into the lesion itself. It was then unfolded and placed on a piece of coordinate paper. The length of the proximal esophageal part of the resected specimen was measured using a graduated ruler (Fig. 4A). After measurement, the specimens were fixed with 10% formalin fixative. After 24 h, additional specimens were obtained from the visible boundary, with an interval of 0.3 cm, until the upper edge. Each tissue was preserved in a wax block, and sections of the wax block were used to make pathological slides. The presence of tumor cells was observed under an electron microscope, and the extent of tumor infiltration was calculated based on the observations made from the slide (Fig. 4B).

### Data analysis

All data were statistically analyzed using SPSS 20.0. Categorical variables were expressed by counts and percentage, and were compared between the two groups using Chi-square tests or Fisher tests. Continuous variables were expressed as mean ± standard deviation and were compared between the two groups using the *t*-test. *P* < 0.05 was considered to indicate statistical significance.

## Results

### General patient characteristics

Between September 2021 and September 2023, a total of 66 patients were randomly divided into an experimental group (*n* = 33) and a routine group (*n* = 33). The patients in the experimental group underwent radical surgery after labeling with titanium clips and carbon nanoparticles, while the patients in the routine group underwent radical surgery without labeling. After surgery, two patients were excluded from the experimental group: one patient was excluded because of contamination of the carbon nanoparticle staining suspension, and one patient was excluded because of peritoneal metastasis. In the routine group, four patients were excluded: one patient had an unresectable tumor, two patients had peritoneal metastasis, and one patient withdrew from the study. After these patients were excluded, 60 patients were included in the final analysis, including 31 patients in the experimental group (24 males and 7 females; average age, 63.43 ± 7.28 years) and 29 patients in the routine group (24 males and 5 females; average age, 61.25 ± 5.66 years). There were no significant differences in the baseline characteristics of the patients, with the exception of the distribution of tumor sites, surgical methods, and reconstruction methods (Table 1).

**Table 1:** General characteristics of the patients (*N* = 60)

	Experimental group (*n* = 31)	Routine group (*n* = 29)	*P*
Gender			
Male	24(77.4%)	24(82.8%)	0.6
Female	7(22.6%)	5(17.2%)	
Age (years)	63.43 ± 7.28	61.50 ± 5.66	0.2
BMI (kg/m2)	22.19 ± 2.22	23.08 ± 2.55	0.15
Siewert type II	31(100.0%)	29(100.0%)	/
Pathological type	31(100.0%)	29(100.0%)	/
Operation time (min)	263.09 ± 14.12	257.22 ± 13.97	0.11
Intraoperative blood loss volume (ml)	172.51 ± 20.34	180.93 ± 21.79	0.12
Length of stay (days)	13.42 ± 1.02	13.37 ± 1.14	0.85
Digestive reconstruction			
Esophagogastrostomy	17(54.8%)	15(51.7%)	0.8
Esophagojejunostomy	14(45.2%)	14(48.3%)	
Negative upper margin	31(100.0%)	29(100.0%)	/
Clinical stage			
0	0(0%)	0(0%)	
I	3(9.7%)	3(10.4%)	
IIA	28(90.3%)	26(89.6%)	0.93
IIB–IV	0(0%)	0(0%)	
cT stage			
T0	13(42.0%)	15(51.7%)	
T1	9(29.0%)	8(27.6%)	0.68
T2	9(29.0%)	6(20.7%)	
cN stage			
N0	13(42.0%)	15(51.7%)	
N1	13(42.0%)	11(37.9%)	0.69
N2	5(16.0%)	3(10.4%)	

### Prediction of the surgical approach

**Fig. 5:**
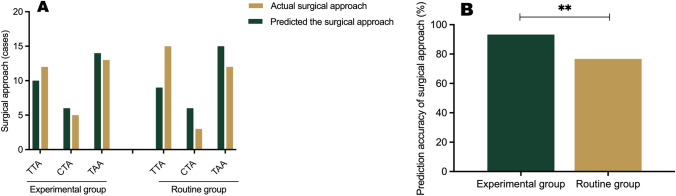
Accuracy of prediction of the surgical approach (**A**). The compliance of three different surgical approaches predicted in the experimental group and the routine group; **B** The overall accuracy of surgical approach prediction in the experimental group and the routine group. *TTA* Transthoracic approach; *TAA* transabdominal approach; *CTA* combined thoracoabdominal approach.**P < 0.05

After the evaluation was completed, the surgical approach was adjusted according to the actual situation during the operation. The accuracy of prediction of the surgical approach was calculated as the number of correct cases divided by the total number of cases in the group. The results showed that the accuracy of prediction for the experimental group and the routine group was 96.8% (30/31) and 75.9% (22/29), respectively (*P* = 0.02). The values imply that the accuracy of prediction was significantly higher in the experimental group than in the conventional group (Fig. 5, Supplementary Table S1).

### Esophageal resection length and tumor infiltration length

**Fig. 6:**
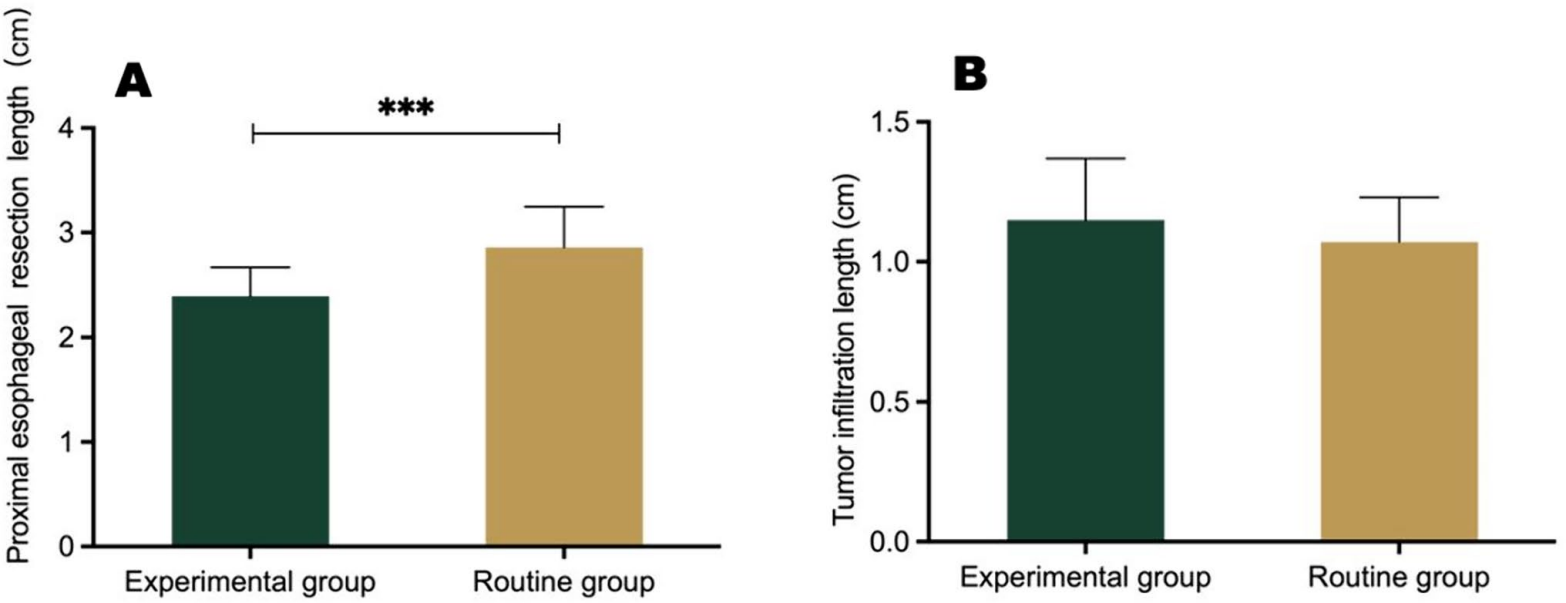
**A** Length of proximal esophageal resection, **B** Tumor infiltration in the experimental and routine groups (***P < 0.001)

The esophageal resection length of the experimental group and the routine group was 2.39 ± 0.28 cm and 2.86 ± 0.39 cm, respectively (*P* < 0.001), and the length of tumor infiltration into the esophagus was 1.15 ± 0.22 cm and 1.07 ± 0.16 cm, respectively (*P* = 0.12). The frozen pathological specimens from both groups were negative for tumor cells (Supplementary Table S2). These results confirm the widespread presence of infiltrating Siewert type II tumors in the entire cohort. Based on the results in the experimental group, it appears that it is possible to resect a shorter esophageal segment while ensuring a negative upper margin with the dual localization technique for prediction of the optimal surgical approach and delineation of the resection margin during the procedure (Fig. 6).

### Postoperative complications

**Fig. 7:**
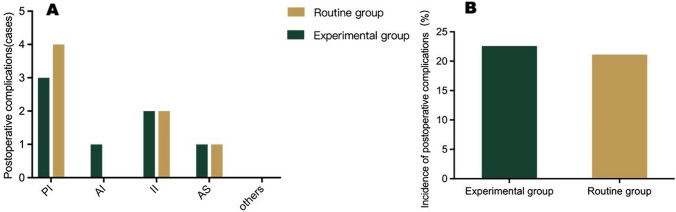
Incidence of postoperative complications in the experimental and routine groups A.The specific postoperative complications of the experimental group and the conventional group; B. the overall postoperative complication rates of the experimental group and the conventional group. *PI* pulmonary infection, *AI* abdominal infection, *II* incision infection, *AS* anastomotic stoma fistula

The overall incidence of complications in the experimental group and the routine group was 22.59% and 24.14%, respectively (*P* = 0.88). In the experimental group, there were three cases of pulmonary infection, one case of abdominal infection, two cases of incision infection, and one case of anastomotic stoma fistula. In the routine group, there were four cases of pulmonary infection, two cases of incision infection, and one case of anastomotic stoma fistula. The incidence of postoperative complications in the experimental group was not significantly higher than that in the routine group and that reported in similar studies(Mine et al. 2022) (Fig. 7, Supplementary Table S3).

## Discussion

The incidence rate of AEG has been increasing every year. However, the unique anatomical location and biological behavior of Siewert type II AEG makes the selection of the surgical approach and determination of the resection range difficult. When planning and performing surgery for early Siewert type II type AEG, it is not possible to accurately determine the extent of invasion based on visual observation and palpation. Moreover, the limitations of traditional staining and labeling methods, as described in the introduction section, seriously affect intraoperative judgment. Surgeons often resect the lesion with a larger margin than required to ensure a negative surgical margin, and there are no standard clinical methods for determining the surgical approach. With regard to the surgical approaches, the transthoracic approach is more invasive and is associated with more complications and a higher postoperative mortality rate. In contrast, the transabdominal approach is associated with more pulmonary complications but a longer survival time (Kurokawa et al. 2013).

In response to the clinical issues regarding the selection of AEG surgical approaches and resection range discussed earlier, this study proposes a dual localization technique involving preoperative EUS-guided labeling with titanium clips and carbon nanoparticles. First, EUS was used to detect tumor invasion in the mucosal layer, submucosal layer, muscular layer, and serosal layer, as well as metastasis to important organs and lymph nodes. As previously published, EUS has great value for determining the scope of tumor invasion and TNM staging (Yong et al. 2013). Second, carbon nanoparticle staining has advantages over traditional staining methods: that is, it has greater stability, longer dyeing time, and no serious side effects. Our team has already reported the advantages of combining EUS technology with carbon nanoparticle staining in preoperative labeling and localization in the treatment of early-stage cancer (Yongwei et al. 2019). This study explored the addition of titanium clips that can be visualized under X-rays for the purpose of visually assessing the extent of invasion of tumor lesions before surgery.

For the selection of surgical approach, as the esophageal hiatus is approximately at the level of the 10th thoracic vertebra, the esophagus enters the abdominal cavity from the thoracic cavity. This study used the positional relationship between the titanium clip and the 10th thoracic vertebral body as the basis for determining the surgical approach: When the titanium clip was placed above the 10th thoracic vertebral body, the surgery was completed through a thoracic approach. When the titanium clip was placed below the 10th thoracic vertebral body, the surgery was completed through an abdominal approach. A combined thoracoabdominal approach was used when the clip was placed between the upper and lower edges of the 10th thoracic vertebral body. The final results show that the accuracy of this method for the prediction of surgical approach was 96.8%, which is significantly better than that of traditional judgment methods.

With regard to determining the length of tissue that needs to be resected above the tumor, the innovative use of titanium clip and carbon nanoparticle dual localization with EUS in this study was found to be useful for accurately detecting the range of invasion, including early tumors. That is, the dual localization technique used here allowed for precise localization of the actual infiltration boundary of the tumors while minimizing the probability of labeling failure. With only nanocarbon labeling, there is a small probability of the particles diffusing beyond the intended region or infiltrating too deep into the tissue, and this could have affected the assessment of the tumor. In addition, the lack of tactile feedback is not conducive to intraoperative positioning. If titanium clips are used alone for labeling, although the position of the titanium clip can be determined through imaging or intraoperative tactile feedback before surgery, there is still a small probability of the titanium clip detaching.

The unique biological features of AEG include its ability to infiltrate and metastasize widely, as well as invade the submucosal lymphatic network of the esophagus (Xiang 2012). The fifth edition of the Japan Gastric Cancer Convention advocates preoperative endoscopic labeling based on biopsy results of the tumor boundary for tumors that invade the esophagus, and examination of intraoperative frozen sections of the cutting edge is also performed to ensure R0 resection (Lei et al. 2018). In this study, continuous pathological sections were used to explore the infiltrating behavior of the tumors. The pathological results confirmed tumor-infiltrating behavior in all 60 cases. In the experimental group, no tumor-positive pathological samples of the upper cutting edge were found, and there was no significant difference in the incidence of postoperative complications compared to the conventional group. This indicates that the dual localization technology employed in the group has sufficient safety and reliability.

The main limitations of this study are its single-center setting and the small sample size. In the future, a multi-center study with a large sample size is required to demonstrate the reliability and effectiveness of the proposed dual localization technique. Another limitation is that there are no follow-up data on postoperative survival time, recurrence rate, and quality of life, as a result of which it is impossible to compare the prognosis of the two groups and determine the potential long-term benefits of this technique. A final limitation of this study is that patients with AED stage IIB and higher were not included because of the risk of titanium clip detachment after neoadjuvant chemotherapy. In the future, we plan to include patients who have gone neoadjuvant chemotherapy and explore if the dual labeling technique with titanium clips and carbon nanoparticles is also beneficial for exploring tumor regression behavior after neoadjuvant chemotherapy in this group of patients, as this could provide a basis for surgical resection after chemotherapy.

## Conclusion

In summary, in patients with Siewert type II AEG, the dual localization technique with carbon nanoparticle staining and titanium clip placement under EUS before surgery can help determine the actual boundary of the lesion based on imaging findings, provide an objective basis for the selection of the surgical approach, and prevent the chances of excessive esophageal resection and tumor-positive margins during surgery. As per the current findings, it is a safe, stable, and reliable method, but further clinical studies with large sample sizes conducted across multiple centers, including randomized control trials, are still needed to demonstrate the effectiveness of this technology.

### Supplementary Information

Below is the link to the electronic supplementary material.

Supplementary file1 (XLSX 91 KB)

Supplementary file2 (XLSX 96 KB)

Supplementary file3 (ZIP 29 KB)

Supplementary file4 (MP4 1560 KB)

## Data Availability

The datasets generated during and/or analysed during the current study are available from the corresponding author on reasonable request.

## Abbreviations

TTA: Transthoracic approach
TAA: Transabdominal approach
CTA: Combined thoracoabdominal approach
PI: Pulmonary infection
AI: Abdominal infection
II: Incision infection
AS: Anastomotic stoma fistula
